# The CHK1 inhibitor Prexasertib is effective against *in vitro* models of aggressive thyroid carcinomas with defective p53 function

**DOI:** 10.3389/fendo.2026.1743111

**Published:** 2026-05-08

**Authors:** Alessandro Manzo, Elisa Stellaria Grassi, Maria Orietta Borghi, Carla Colombo, Luca Persani, Valentina Cirello

**Affiliations:** 1 Department of Medical Biotechnology and Translational Medicine, University of Milan, Milan, Italy; 2 Department of Endocrine and Metabolic Diseases, Istituto Auxologico Italiano IRCCS, Milan, Italy; 3 Immunorheumatology Research Laboratory, IRCCS Istituto Auxologico Italiano, Milan, Italy; 4 Dipartimento di Scienze Cliniche e di Comunità, Dipartimento di Eccellenza 2023-2027, University of Milan, Milan, Italy; 5 Department of Pathophysiology and Transplantation, University of Milan, Milan, Italy

**Keywords:** CHK1, DNA damage response, Prexasertib, thyroid cancer, *TP53*, tyrosine kinase inhibitors

## Abstract

**Introduction:**

Tyrosine kinase inhibitors represent the most effective and long-lasting treatment currently available for aggressive thyroid cancers, but in some patients the response is poor or absent. Recently, we demonstrated that the response to Lenvatinib is associated with alterations in *TP53* gene or protein. Aiming to find a novel therapeutic strategy for aggressive thyroid cancers, we investigated *in vitro* the DNA damage response pathway, where p53 plays a crucial role, and tested its inhibition through synthetic lethality or stress sensitization strategies.

**Methods:**

The DNA damage response has been characterized in a panel of p53-defective or proficient thyroid cancer cell lines both at basal level and in response to Doxorubicin, a DNA damaging agent, by western blot, immunofluorescence and MTT-based cell viability assay. We then evaluated the effect of a selective CHK1 kinase inhibitor, Prexasertib, either alone or in combination with Doxorubicin. Cell cycle variations and cell death were evaluated by flow-cytometry.

**Results:**

The p53-defective thyroid cancer cells showed a higher degree of genomic instability and, in response to Doxorubicin, activated not only ATM/CHK2 but also ATR/CHK1 to bypass p53 and induce DNA repair. We found that Prexasertib was more effective in p53-defective cells, since it significantly reduced tumor cell proliferation due to replicative failure and cell death. Moreover, Doxorubicin potentiated the Prexasertib effects in p53-defective thyroid cancer cells, yet at the lowest doses.

**Conclusions:**

This study unravels the potential of Prexasertib as a novel treatment option for aggressive thyroid cancers p53-defective and poorly responsive to tyrosine kinase inhibitors.

## 1 Introduction

Thyroid cancers (TCs) of follicular origin include well-differentiated thyroid cancers, generally responsive to conventional therapies, surgery and radioactive iodine therapy, as well as more aggressive forms, namely poorly differentiated and anaplastic thyroid cancers, which represent a major clinical challenge (1, 2). Aggressive forms of TCs show limited benefit from conventional therapies and are typically treated with multi-tyrosine kinase inhibitors (TKIs), such as Lenvatinib, or with drugs against molecular targets (3, 4). TKIs represent the most effective treatment for advanced TC (3, 4), but in some patients the response is poor or absent, due to primary or acquired resistance mechanisms (4). Thus, an alternative therapeutic approach for aggressive and TKI-resistant TCs is still awaited.

In accordance with findings in hepatocellular carcinoma and non-small cell lung cancer (5, 6), we recently demonstrated that *TP53* gene or p53 protein alterations in advanced TC are predictors of poor response to Lenvatinib treatment, associated with worse progression-free survival and overall survival rates (7). *TP53* alterations are frequent in aggressive TCs (8, 9) and can cause genomic instability (9, 10), favoring the accumulation of unresolved DNA damages. When p53 function is lost, these potentially lethal genomic lesions can be repaired through kinases involved in DNA damage response pathway (DDR), like ATR/CHK1 (*CHEK1* identifies the gene name, while CHK1 refers to the encoded protein) and ATM/CHK2, involved in repairing DNA single- or double-strand breaks (DSBs) respectively, thus allowing cancer cells survival (11).

Specific inhibitors targeting these DDR kinases have been developed and are already being investigated in clinical trials for the treatment of solid and hematological tumors (11, 12), but not for TCs. Therefore, the loss of p53 functionality can be exploited to make tumor cells vulnerable by inhibiting a DDR kinase activated in aggressive TCs. There are two possible therapeutic strategies that can lead to cancer cell death. The first is synthetic lethality, which can be achieved by inhibiting a DDR molecule that compensates for the loss of the vital function of p53. The second is stress sensitization, which involves combining the inhibition of a DDR molecule with the addition of a chemotherapy agent that induces genomic damage in p53-defective cancer cells (11, 13).

The aim of the study was to screen the main kinases of the DDR pathway to find a suitable molecular target to exploit for the treatment of p53-defective aggressive TC.

## 2 Materials and methods

### 2.1 Cells cultures

TPC-1 (human papillary thyroid carcinoma, PTC), FRO (human anaplastic TC, ATC) and NTHYORI 3-1 (human primary thyroid follicular epithelial cells) cell lines were grown in RPMI with 2mM Glutamine (Euroclone, Pero, Italy), SW1736 (human ATC), HTC/C3 (human metastasis of poorly differentiated TC, PDTC), and B-CPAP (human PDTC) in DMEM (Gibco-Thermo-Fisher Scientific, USA), while IHH-4 (human ATC) in DMEM/RPMI. *TP53* inactivating mutations affect SW1736, B-CPAP and HTC/C3, while *TP53* silencing occurs in FRO. In contrast, *TP53* is wild-type in TPC-1 and IHH-4 (14). All cell lines, STR-authenticated and mycoplasma-tested, were cultured in media supplemented with 10% FBS (Sigma-Aldrich, Missouri, USA) and penicillin-streptomycin (Sigma-Aldrich) at 37 °C, 5% CO_2_. Experiments were performed up to the 17^th^ passage.

### 2.2 Cell treatments and proliferation

Doxorubicin hydrochloride (DX) (Divbio Science, Netherlands) and CHK1-inhibitor Prexasertib dimesylate (PX) (Fisher Scientific, NewHampshire, USA) were diluted in distilled water and stored at -80 °C. For combined treatments, PX was added 30 minutes before DX treatment as already reported (15, 16). Proliferation was assessed by MTT assay (MedchemExpress, New Jersey, USA), in 96-well plates, with 2x10^3^ cells per well, as previously described (17). All experiments and Inhibitory Concentrations (ICs) were assessed after 48 hours of treatment.

### 2.3 Western blot

2x10^5^ cells/well were seeded in 6-well plates. After treatment they were lysed with 62.5 mM Tris-HCl 1% SDS buffer (pH 6.8) with protease and phosphatase inhibitors (cOmplete Tablets Mini and PhosStop, Roche, Switzerland). Sonicated samples were centrifuged at 11.000g for 15 minutes at 4 °C and supernatant was quantified by BCA assay (ThermoFisher, Massachusetts, USA). We aimed at loading 20 μg of protein in 3-8% NuPAGE Tris-Acetate protein gels (ThermoFisher) for high-molecular weight proteins, otherwise in 4-12% NuPAGE Bis-Tris Gels in MOPS buffer (ThermoFisher). In some cases, due to high cytotoxicity, less than 20 μg of proteins were retrieved and loaded. HiMark^TM^ Pre-stained Protein Standard (ThermoFisher) was used for high molecular weight proteins, otherwise Rainbow Molecular Weight Marker (Cytiva, Washington, USA). Proteins were transferred with iBlot Blotting System (ThermoFisher) according to manufacturer instructions. Nitrocellulose membranes were blocked for 1 hour at room temperature with 5% BSA in TBS-T and incubated overnight at 4 °C on a shaker with the primary antibody (β-actin diluted 1:5.000, otherwise 1:1.000). Primary antibodies used were p-ATM (S1981), p-ATR (S428), p-CHK1 (S345), p-CHK2 (T68), p-H2AX (Ser139), pCDC25c, tot-ATM, tot-ATR, tot-CHK1, tot-CHK2, PARP, Caspase3, vinculin (Cell Signaling Technology, Massachusetts, USA) and β-actin (BD Biosciences, New Jersey, USA). Secondary antibody diluted 1:5.000 (either anti-rabbit or anti-mouse HRP-conjugated antibodies, Cell Signaling), was incubated for 1 hour at room temperature on shaker. Detection was performed using Westar Supernova ECL Substrate (Cyanagen, Bologna, Italy) with Azure Biosystem C400 camera (Azure Biosystems, California, USA). Band intensity was quantified with FIJI software, v.2.3.0 (18). After the acquisition of phosphorylated forms, membranes were stripped for 30 minutes before blotting total forms. Membranes were cut by molecular weight owing to the number of samples.

### 2.4 Immunofluorescence

2x10^5^ cells/well were seeded in 6-well plates on glass slides pre-coated with Poly-L-lysine (Merck, Massachusetts, USA) for 1 hour at 37 °C. After treatment, samples were fixed 10 min in 4% Paraformaldehyde (Santa Cruz biotechnology, Texas, USA) and permeabilized 10 minutes with 0.2% Triton-X. After 1 hour blocking with 5% BSA/PBS, overnight incubation at 4 °C with primary antibody anti-γH2AX (Cell Signaling) diluted 1:400 was performed. Secondary antibody incubation with anti-rabbit Alexa-Fluor488 conjugated (ThermoFisher) diluted 1:1.000 was performed for 1 hour in the dark. After PBS washing, samples were mounted on microscope slides with DAPI Vectashield Hard-Set mounting medium (Vector, California, USA). Images were acquired with Nikon EclipseTi-E inverted microscope with IMA10X Argon-ion laser System (Melles Griot, California, USA). Images were acquired with CFI Plan Apo VC 20X (Nikon) objective in one single Z-scansion by selecting the mean focus plane in the DAPI channel. At least 3 randomly selected fields and 100 nuclei per replicate were analyzed with FIJI software, v.2.3.0 (18). The γH2AX signal intensity was measured on randomly selected nuclear regions of interest (ROI) defined in the DAPI channel. NTHYORI 3–1 cell line mean value was used as an internal control for each replicate.

### 2.5 ELISA assay

2x10^5^ cells/wells were seeded in 6-well plates, lysed, and tot-CHK1 and p-CHK1(S296) levels were quantified by ELISA assay (RayBio^®^ Phospho-Chk1 (Ser296) and Total Chk1 ELISA kit). Colorimetric absorbance at 450 nm was read with ELx800 Absorbance Microplate Reader (BioTek, Agilent, USA).

### 2.6 Apoptosis and cell cycle

5x10^4^ cells/wells were seeded in 6 well plates. After treatment, cells were harvested by trypsinization and washed with cold PBS. For cell cycle analysis, cells were fixed with 70% ethanol/PBS for 1 hour at 4 °C. After centrifugation at 670g for 5 min at 4 °C, cell suspension was stained with FxCycle^TM^ PI/RNase Staining Solution (ThermoFisher). Ten thousand events per sample were acquired by FACSLyric flow cytometer (Becton Dickinson, Erembodegem, Belgium) at 532nm excitation, following manufacturer indications. Cell death was evaluated by AnnexinV Alexa FluorTM 488 & Propidium Iodide (Dead Cell Apoptosis Kit, ThermoFisher) staining, according to manufacturer instructions. Analyses were performed by BD Software (Becton Dickinson).

### 2.7 Statistical analysis

Data were presented either as mean (± SEM) or median (± IQR) of at least 3 independent experiments based on the Shapiro-Wilk normality test. Parametric One-way ANOVA test was applied for normally distributed data, otherwise non-parametric Kruskal-Wallis’s test was used. Parametric Two-way ANOVA test was applied for combined treatments. For multiple comparison analyses, parametric Dunnett’s and Tukey’s tests were used, otherwise Dunn’s test was performed. Statistical significance was set at p<0.05 with p-values: *p<0.05; **p<0.01; ***p<0.001. Analyses were performed with GraphPad Prism9 software.

## 3 Results

### 3.1 Thyroid cancer cell lines display intrinsic DNA damage foci

We first evaluated the presence of unrepaired DNA damage, resulting from genomic instability, in our panel of TC cell lines. The phosphorylated form (Ser139) of histone γH2AX, a marker of DNA damage, was assessed by immunofluorescence analysis. As expected, p53-defective TC cells (FRO, B-CPAP, HTC/C3) exhibited higher basal levels of DNA damage compared to the p53-proficient ones (TPC-1 and IHH-4), except for SW1736 cells showing γH2AX expression levels similar to those of p53-proficient cells (**Supplementary Figures 1A, B**).

### 3.2 P53-defective TC cell lines show variable expression levels of DNA damage response kinases

Considering that DNA damage was intrinsically more prevalent in p53-defective TC cell lines compared to p53-proficient ones, we further investigated *in vitro* the DNA damage response (DDR) pathway, where p53 plays a central role (**Supplementary Figure 2A**). By comparing the basal expression levels of key DDR kinases - ATR, CHK1, ATM and CHK2 - we found that p53-defective TC cell lines exhibited greater variability in DDR proteins expression compared to the p53-proficient ones, except for ATR kinase, which was consistently expressed across all TC cell lines (**Supplementary Figure 2B**). CHK1 was expressed at high levels in SW1736 and B-CPAP cells, whereas ATM was poorly expressed in SW1736 cells despite the high levels of the downstream kinase CHK2. The latter was notably low in FRO cells (**Supplementary Figure 2B**).

### 3.3 ATR/CHK1 axis is involved in the signaling of DNA DSBs in p53-defective TC cell lines

**Figure 1:**
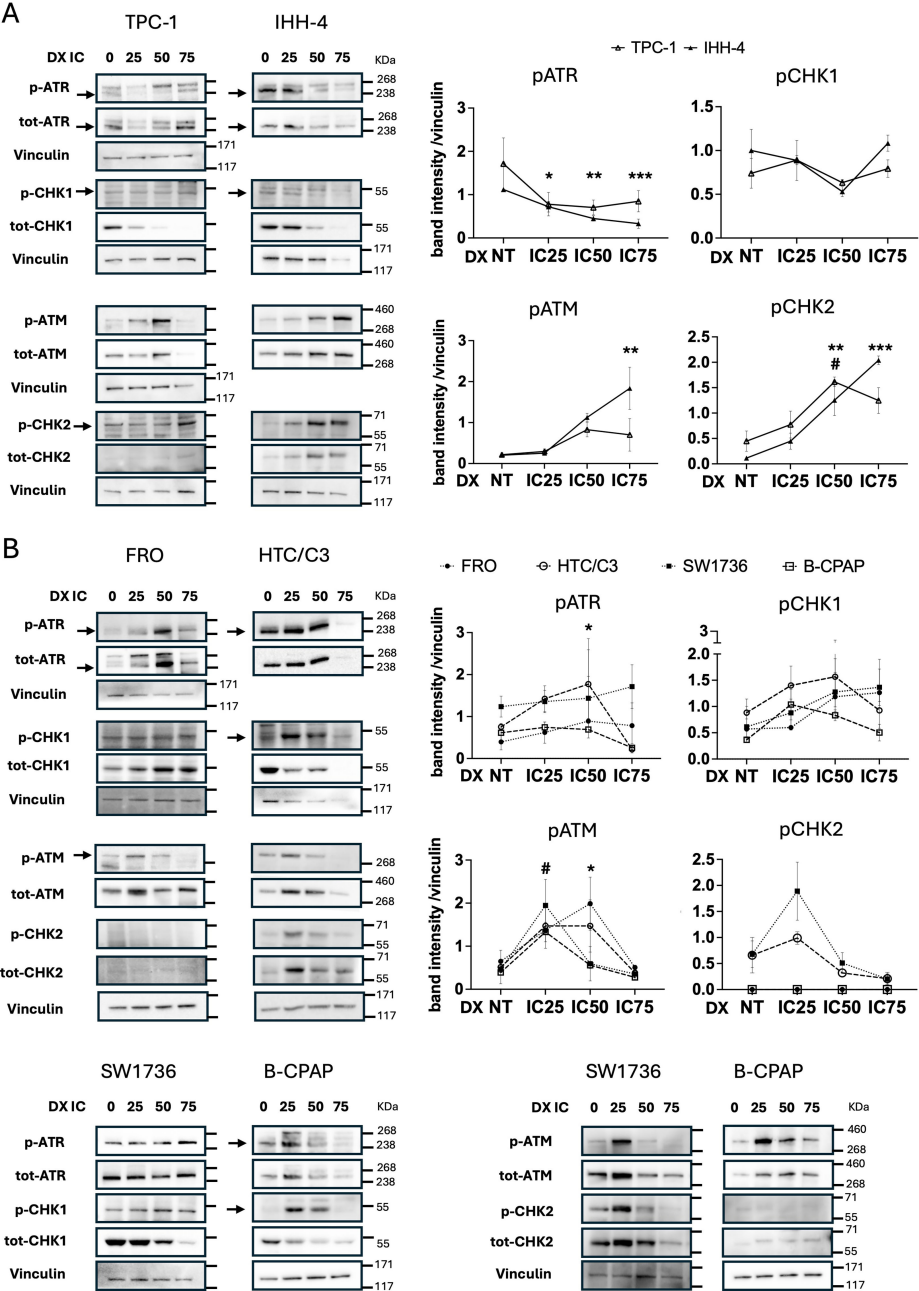
p53-defective TC cell lines signal DNA DSBs via ATR/CHK1 activation. TC cell lines were assayed by western blot after treatment with increasing Doxorubicin doses [IC25, IC50, IC75] for 48 hours. Densitometric data (normalized over Vinculin) and their representative bands show DDR kinases phosphorylation (and total form) in p53-proficient **(A)** and p53-defective **(B)** TC cell lines. In IHH-4 and HTC/C3 cells, bands corresponding to total CHK1 and vinculin were markedly reduced at the highest DX dose, due to the low amount of protein (<20 μg) recovered and loaded as a result of high cytotoxicity. HiMark Pre-stained Protein Standard (ThermoFisher) was used. Parametric One-Way ANOVA followed by Dunnett’s test were performed and data expressed as mean ± SEM. For ATR and CHK1 of the FRO cell line, non-parametric Kruskal-Wallis followed by Dunn’s test were used and data expressed as median ± IQR. Symbols * or # indicate significant p value (*/#p<0.05; **/##p<0.01; ***/###p<0.001) for different cell lines: * for IHH-4 and FRO, # for TPC-1 and HTC/C3. p, phosphorylated; tot, total; NT, non-treated; prof, proficient; def, defective.

We next aimed to characterize DDR kinases activation in our TC cell lines upon inducing DNA DSBs with Doxorubicin (DX) treatment. Dose-response curves showed that, as expected, p53-defective TC cells required higher DX doses to reach the Inhibitory Concentration IC50 compared to the p53-proficient ones (**Supplementary Figure 3**). Next, we treated TC cell lines with increasing IC doses of DX and performed western blot analysis to compare the changes in activating phosphorylation of DDR kinases (pATM, pCHK2, pATR, and pCHK1). In p53-proficient TC cell lines (TPC-1 and IHH-4), both pATM (Ser1981) and pCHK2 (Thr68) levels were increased, whereas no activation was observed for pATR (Ser428) and pCHK1 (Ser345) kinases (**Figure 1A**). On the other hand, in p53-defective TC cell lines (FRO, SW1736, HTC/C3, and B-CPAP), DX treatment not only induced activation of pATM and pCHK2 kinases, but also that of pATR and pCHK1 kinases (**Figure 1B**). Interestingly, as DX doses increased, the total form of CHK1 decreased while the other DDR kinases remained stable in all TC cell lines tested (**Figures 1A, B**).

### 3.4 TC cell lines are sensitive to the CHK1 inhibitor Prexasertib

Considering the overall preliminary data, we decided to test Prexasertib (PX), a second-generation ATP-competitive inhibitor selectively targeting CHK1 and CHK2 kinases (Ki= 0.9 nM and Ki= 8 nM, respectively) (19). First, we treated TC cell lines with escalating doses of PX (0-20 μM) and observed a dose-dependent decrease in cell viability across all TC cell lines, with a maximal effect (IC100) at concentrations ranging from 23.9 nM to 40.1 nM, except for B-CPAP cells, which showed very limited sensitivity (**Supplementary Figure 4**). As expected, the sensitivity to PX was higher in p53-defective cells than in the p53-proficient ones (mean IC50 6.4 nM vs 13.1 nM, respectively) (**Supplementary Figure 4**).

### 3.5 PX specifically inhibits CHK1 kinase in TC cell lines

To assess whether PX specifically inhibited CHK1 kinase in TC cell lines, we evaluated the phosphorylation levels of pCHK1 (Ser296) by ELISA assay. We treated TC cell lines with the mean IC values for each group of sensitivity, as shown in **Supplementary Figure 4**, and found that pCHK1 (Ser296) levels were mostly depleted in FRO, SW1736, HTC/C3 and IHH-4 cell lines, yet at the lowest PX dose (4.1 nM), whereas they were highly decreased in B-CPAP and TPC-1 (**Supplementary Figure 5A**). The total form of CHK1 was reduced by PX treatment in a dose-dependent manner in all TC cell lines (**Supplementary Figure 5B**).

### 3.6 PX effect on TC cell viability is potentiated by DX according to stress sensitization strategy

**Figure 2:**
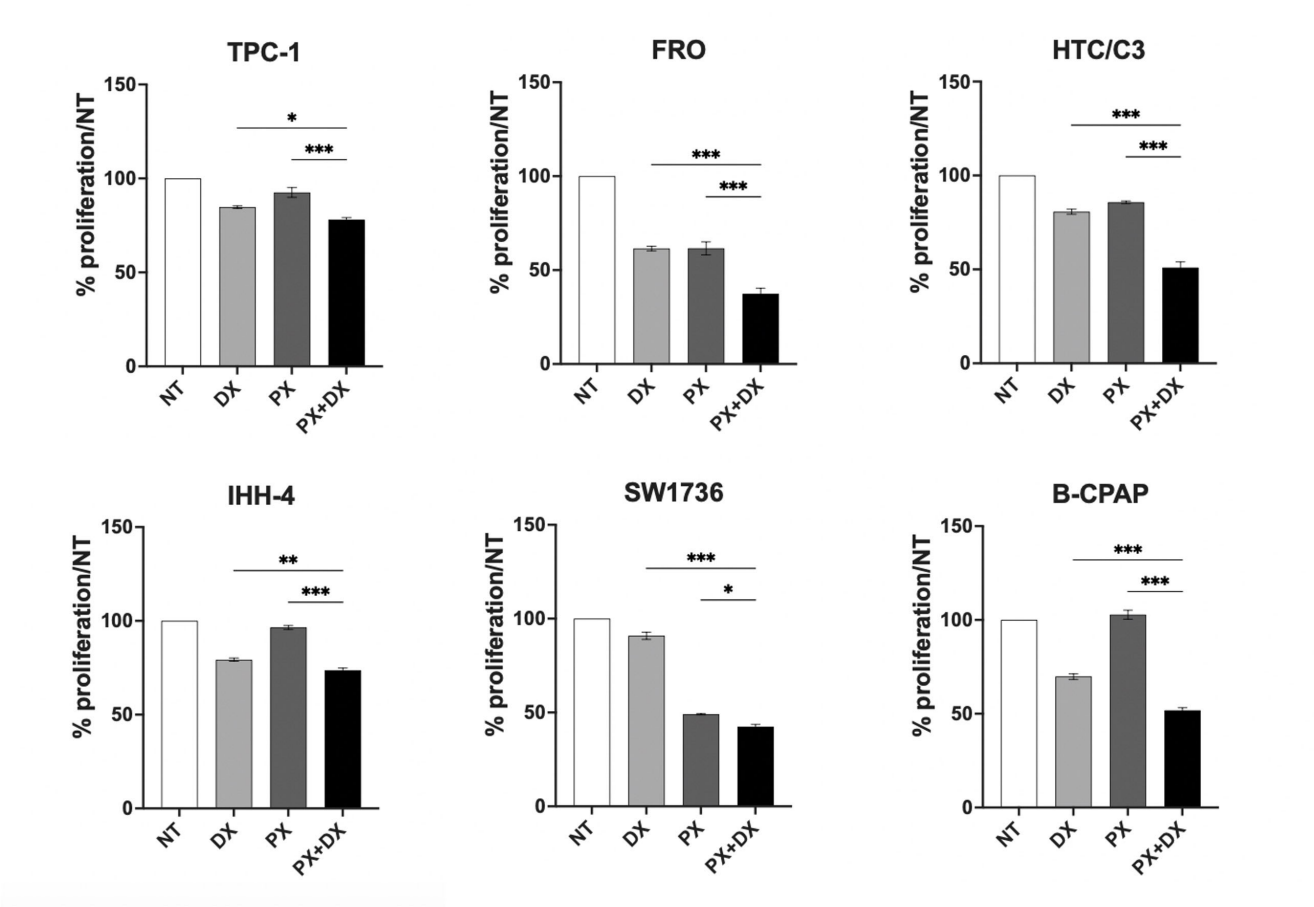
PX alone or with DX reduces TC cell lines viability. After treatment with PX (4.1 nM) and DX (IC25) alone or combined for 48 hours, cell proliferation was expressed as percent of cells vs NT sample by mean ± SEM. Kruskal-Wallis test followed by Dunn’s test were used and data expressed as median ± IQR (*p<0.05; **p<0.01; ***p<0.001). NT, non-treated; DX, Doxorubicin; PX, Prexasertib.

Considering that the combination of DNA-damaging agents is known to enhance the effects of DDR inhibitors, we aimed to evaluate the effect of PX on TC cells in combination with DX. By combining the lowest doses of both DX and PX, we found that TC cell viability was significantly decreased compared to single treatments, particularly in p53-defective TC cell lines (**Figure 2**). Interestingly, B-CPAP cell viability, although unaffected by PX alone, decreased more with the combined treatments than with DX alone (**Figure 2**).

### 3.7 PX induces DNA damage and leads to a reduction in the inhibitory phosphorylation of CDC25c, a direct target of CHK1

**Figure 3:**
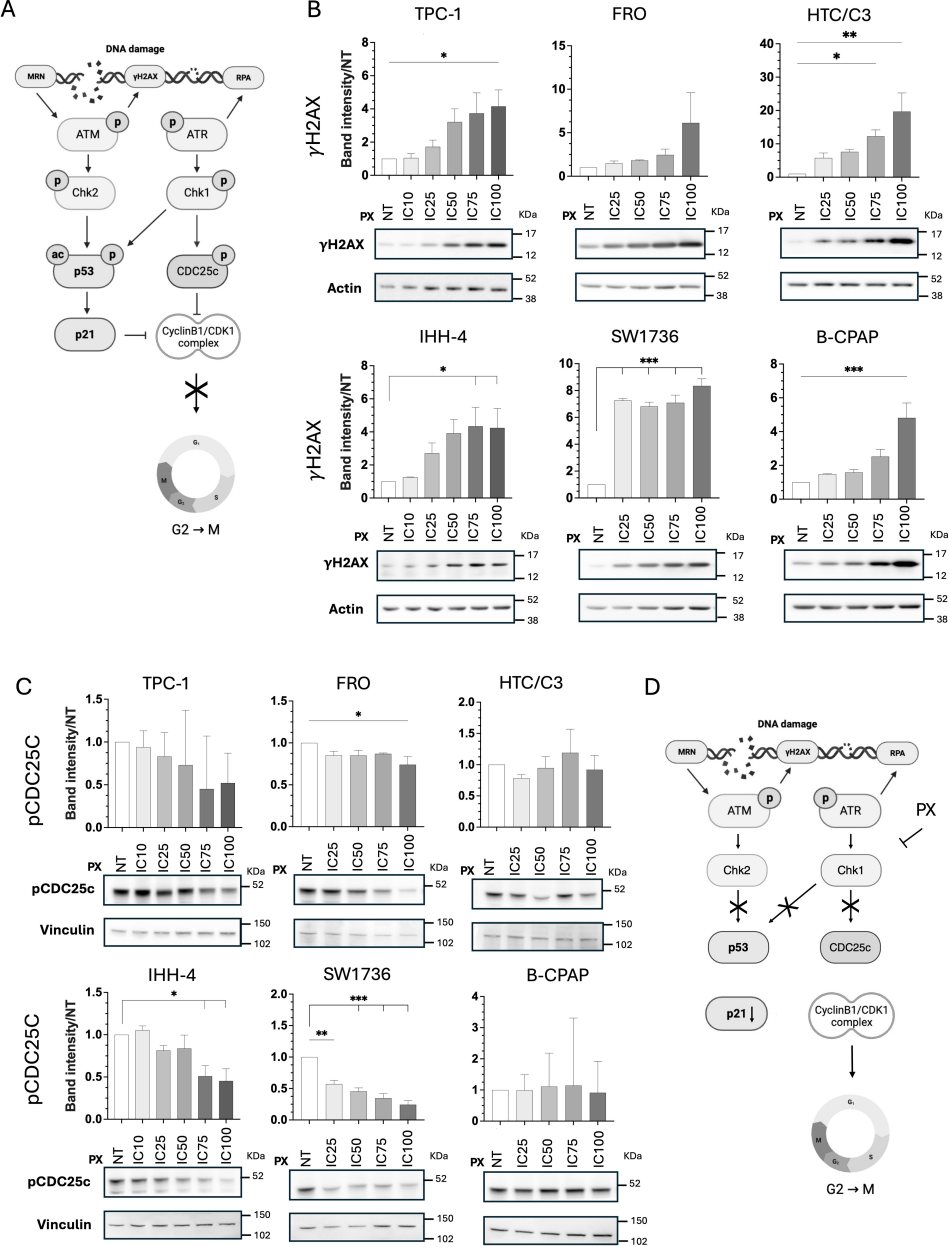
PX induces DNA damage in all TC cell lines and mainly reduces CDC25c inhibitory phosphorylation. **(A)** Scheme of DDR pathway created with https://BioRender.com (Agreement number: DE632A14-0002). **(B)** Western blot images of DNA damage marker γH2AX expression in TC cell lines treated with PX mean ICs and densitometric quantification normalized to both Actin and NT. DNA damages increase upon PX treatments in all TC cells. **(C)** Western blot images of CHK1 direct target pCDC25c expression after PX treatment and densitometric quantification normalized to both Vinculin and NT. Most TC cells show reduced CDC25c inhibitory phosphorylation. Cells were treated for 48 hours **(B, C)**. Rainbow Molecular Weight Marker (Cytiva) was used. **(D)** Scheme of DDR pathway alteration upon PX treatment. Kruskal-Wallis test followed by Dunn’s test were used and data expressed as median ± IQR (*p<0.05; **p<0.01; ***p<0.001). NT, non-treated; p, phosphorylated.

We next investigated the downstream effects of PX by performing western blot analysis for γH2AX and CDC25c phosphorylation levels. Upon DNA damage, phosphatase CDC25c is inhibited by pCHK1 phosphorylation on Ser216, thus preventing the stabilization of the Cyclin B1-CDK1 complex. This pro-mitotic complex can also be inhibited by p53/p21 to eventually block cell cycle progression towards the G2/M phase and induce DNA repair (**Figure 3A**).

First, we found that DNA DSBs, marked by the presence of γH2AX, increased in a dose-dependent manner upon PX treatment in all TC cell lines, regardless of their p53 functional status (**Figure 3B**).

Then we evaluated the phosphorylation levels of phosphatase CDC25c that, upon DNA damage, is inhibited by pCHK1 phosphorylation on Ser216. Upon CHK1 inhibition, due to increasing PX doses, pCDC25c levels gradually decreased in TC cell lines, except in B-CPAP and HTC/C3 (**Figure 3C**). The inhibition of CHK1 and its downstream target, CDC25c, by PX is summarized in **Figure 3D**.

### 3.8 PX-induced DNA damage can lead to S phase block in p53-defective TC cell lines

Then, we proceeded to investigate how the DNA damage induced by PX and the downstream activation of CDC25c could affect the cell cycle in TC cell lines. To compare cell cycle variations in TC cell lines by flow cytometry, we treated cells with fixed PX doses, selecting those that induced IC25 and IC75 in the most PX-sensitive TC cells (4.1 nM and 10.6 nM, respectively).

**Figure 4:**
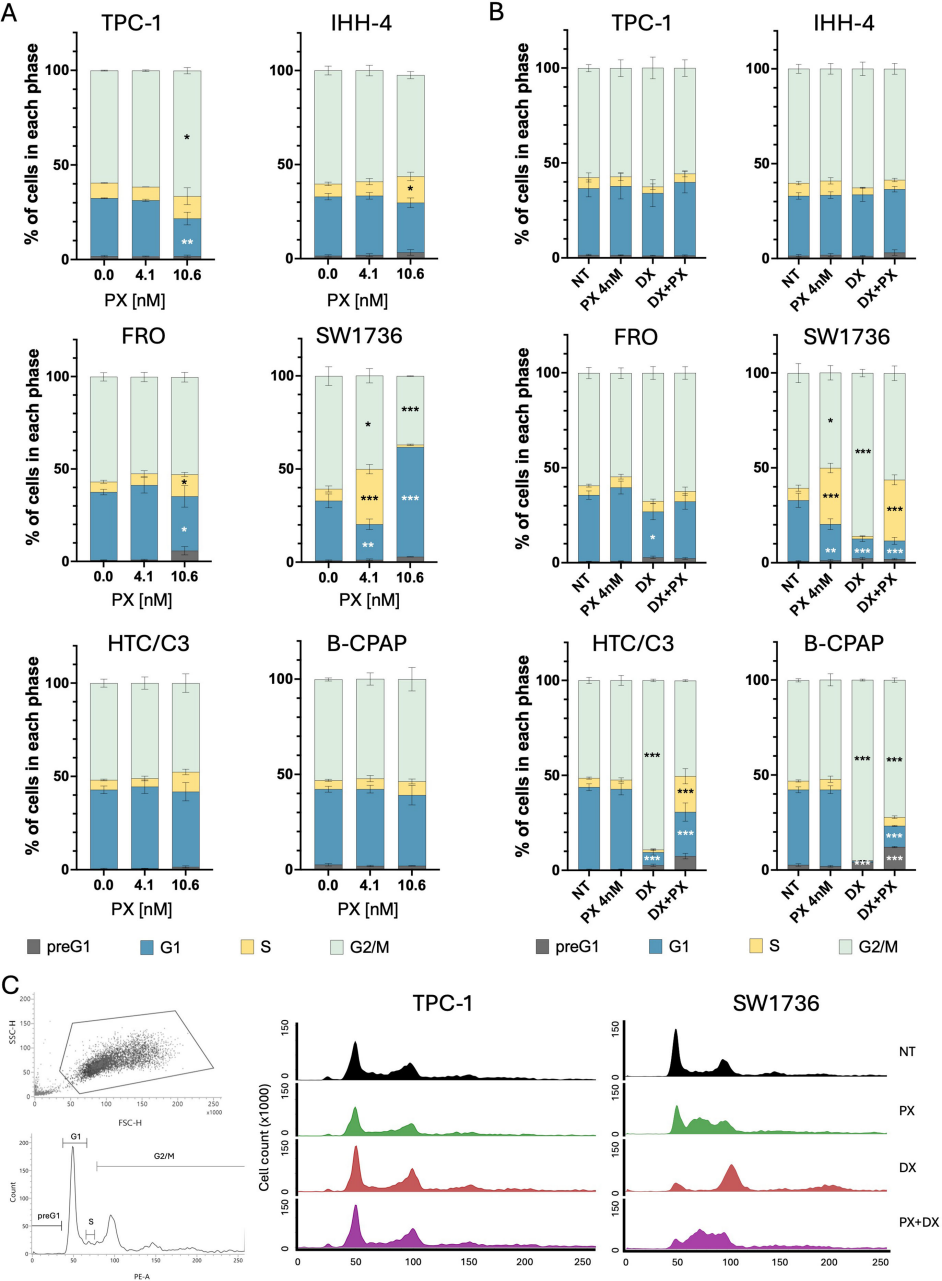
Cell cycle analysis of TC cells treated with PX alone or PX with DX. **(A)** Stacked bar chart of cells percentages in each cell cycle phase upon PX treatment for 48 hours, showing an increase of cells in G1 or S phases in FRO, SW1736, HTC/C3 and IHH-4, while TPC-1 cells increased in G2/M phase. **(B)** Stacked bar chart of cell percentages in each cell cycle phase upon DX and PX single or combined treatments, showing an increase of cells in S phase in HTC/C3 and B-CPAP upon DX+PX treatment. **(C)** Cell cycle analysis via flow cytometry, showing on the left the adopted gating strategy based on physical parameters (FSC/SSC) and below the representative PI histogram showing cell cycle phase quantification (preG1, G1, S and G2/M), on the right a stacked overlay of PI intensity across treatments: NT, PX, DX and DX+PX. Two-Way ANOVA followed by Tukey’s tests were applied and data expressed as mean ± SEM (*p< 0.05; **p<0.01; ***p< 0.001). PX, Prexasertib; DX, Doxorubicin; NT, non-treated. PI, propidium iodide; FSC, Forward SCatter; SCC, Side SCatter.

TPC-1 cells showed an increase in the G2/M phase cell population upon 10.6 nM PX treatment, possibly due to the pre-mitotic cell cycle blockade fostered by p53 in response to PX-induced DNA damage. In contrast, when p53 is defective, PX induced an increase in the number of cells in the S phase as observed for FRO, SW1736 and HTC/C3 cell lines, and in G1 only for SW1736, compared to the non-treated samples (**Figure 4A**). Interestingly, the p53-proficient cell line IHH-4 also showed a significant increase in the number of cells in S phase (**Figure 4A**). Overall, these findings suggest DNA replication failure.

When a DX concentration as low as IC25 was added to 4.1 nM PX, the induction of further DNA damage by DX not only significantly increased the number of S phase cells in the HTC/C3 cell line, but also led to a significant increase in the pre-G1 phase cell population in B-CPAP cells and, tendentially, in HTC/C3 cells, compared to the non-treated samples, suggesting the presence of cell death (**Figure 4B**).

### 3.9 PX induces cell death in p53-defective TC cell lines either alone or in combination with DX

**Figure 5:**
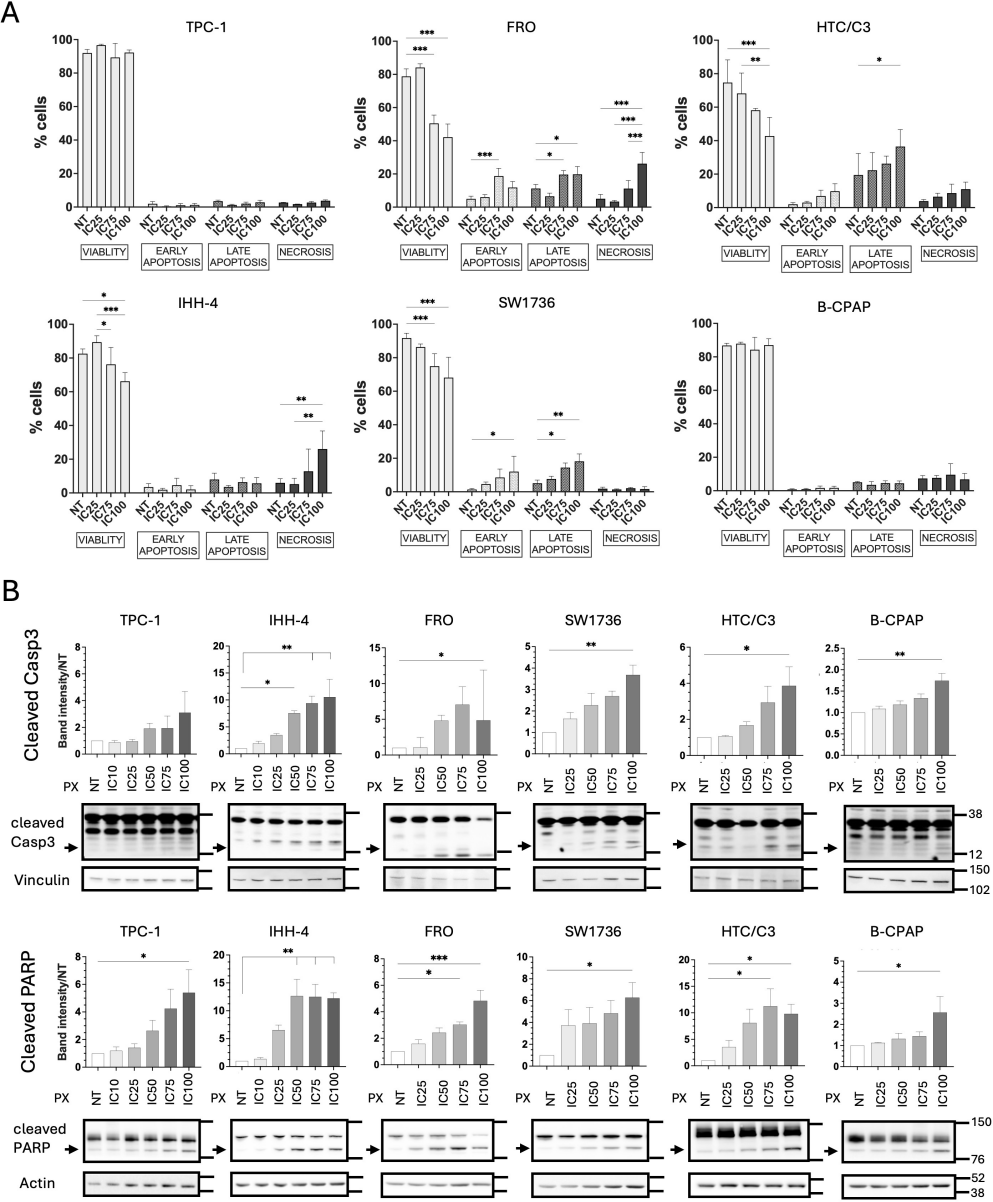
PX induces cell death in aggressive TC cell lines. Upon PX treatments p53-defective TC cells showed dose-dependent increase of mortality **(A)** as well as of apoptotic markers **(B)**. All TC cell lines were treated for 48 hours with mean ICs of PX, relative to each TC group of p53 functionality, and with IC10 PX in p53-proficient cells. In **(A)**, flow cytometry data of AnnexinV/PI-staining shown as percent of cells at each stage (viability, early or late apoptosis, necrosis). Two-Way ANOVA followed by Tukey’s test were applied and data expressed as mean ± SEM. In **(B)**, western blot images of cleaved Caspase3 and PARP expression and densitometric quantification were normalized to both the housekeeping (Vinculin or Actin) and to NT. Rainbow Molecular Weight Marker (Cytiva) was used. Kruskal-Wallis followed by Dunn’s test were applied and data expressed as median ± IQR (*p<0.05; **p<0.01; ***p<0.001). NT, non-treated. PX, Prexasertib.

Finally, we evaluated cell death induction in TC cell lines upon PX treatment, either alone or in combination with DX. Performing flow cytometry analysis with Annexin V/PI staining, we found that increasing doses of PX were able to induce both apoptosis and necrosis in FRO, only apoptosis in SW1736 and HTC/C3 cells, and only necrosis in IHH-4 cells. In contrast, no mortality effect was detected by flow cytometry in B-CPAP and TPC-1 cells (**Figure 5A**). However, western blot analysis showed an increase in PARP and Caspase 3 cleavages as PX doses increased in all p53-defective cells (**Figure 5B**). Interestingly, the apoptotic markers were found to be at least tendentially increased in both p53-proficient cell lines (TPC-1 and IHH-4) upon the highest PX dose (**Figure 5B**).

**Figure 6:**
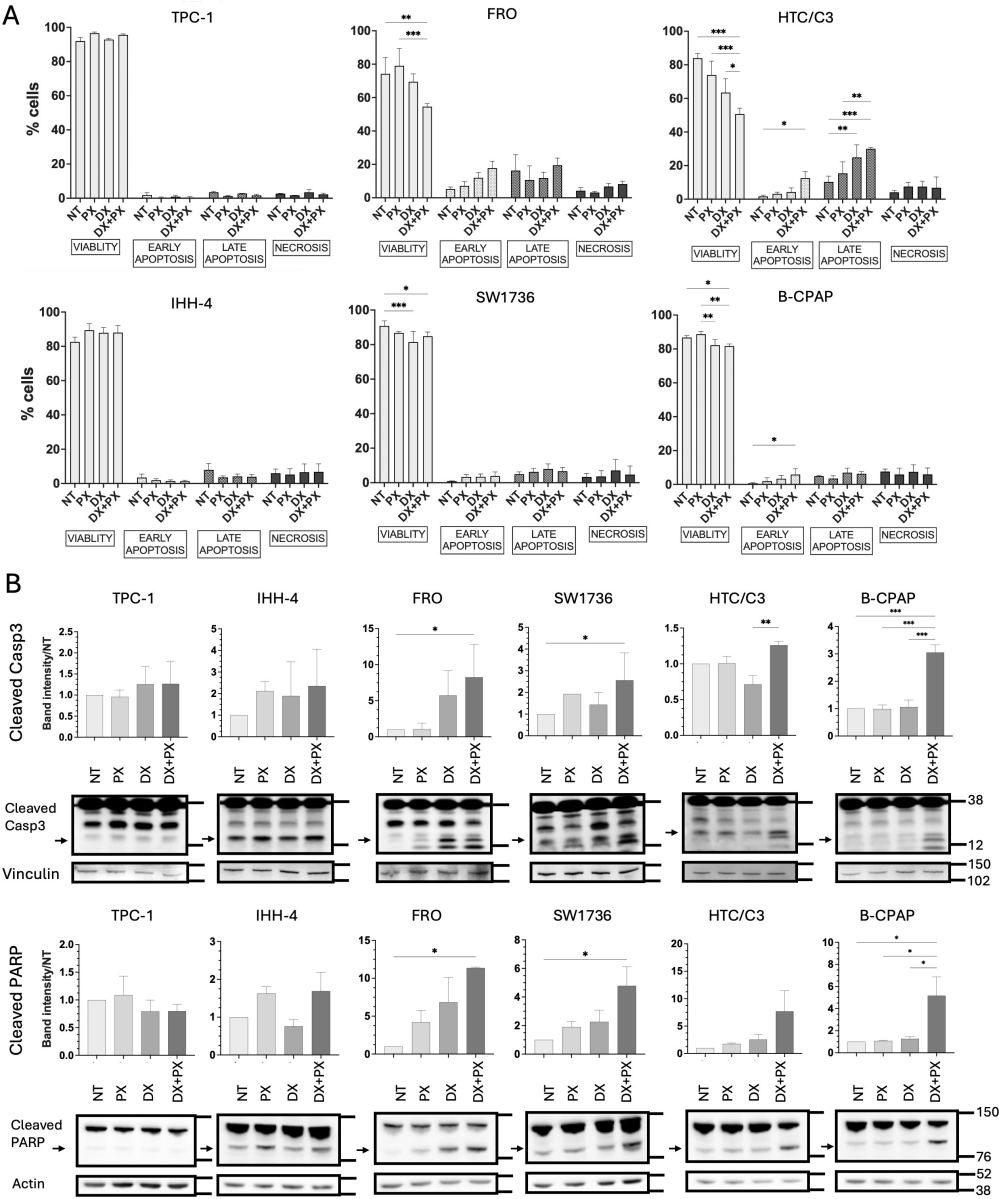
PX and DX combined treatments increase cell death in p53-defective TC cells. Flow cytometry with AnnexinV/PI-staining **(A)** and western blot for cleaved Caspase3 and PARP **(B)** of TC cell lines treated for 48 hours with PX (4.1 nM) and DX (IC25) lowest concentrations, either alone or in combination. In **(A)**, DX+PX increased cell death in all p53-defective TC cell lines. Flow cytometry data are shown as cell percentages at each stage (viability, early or late apoptosis, necrosis). Two-Way ANOVA followed by Tukey’s test were applied and data expressed as mean ± SEM. In **(B)**, DX+PX increased intrinsic apoptosis markers in all p53-defective TC cell lines. Western blot images of cleaved Caspase3 and PARP expression and densitometric quantification normalized to both the housekeeping (Vinculin or Actin) and to NT. Rainbow Molecular Weight Marker (Cytiva) was used. Kruskal-Wallis followed by Dunn’s tests were applied and data expressed as median ± IQR (*p<0.05; **p<0.01; ***p<0.001). PX, Prexasertib; DX, Doxorubicin; NT, non-treated.

Notably, when DX IC25 was added to 4 nM PX to treat TC cells, it increased B-CPAP apoptosis compared to PX single treatment (**Figure 6A**) and promoted higher cleavage of both apoptotic markers (**Figure 6B**), suggesting that the addition of DX to PX-induced DNA damage becomes deleterious for this cell line. Moreover, in the other p53-defective TC cells, the combination of DX and PX induced an increase in both apoptotic and necrotic cells compared to PX single treatment, as evidenced in p53-defective cell lines by the significant reduction in total cell viability (**Figure 6A**), which is confirmed by the significantly increased levels of apoptotic markers (**Figure 6B**).

## 4 Discussion

Novel therapeutic approaches are urgently required for patients with aggressive thyroid cancer (TC) showing resistance to conventional therapies and tyrosine-kinase inhibitors (TKIs) (20). Aiming to identify a novel molecular target for TC treatment, we investigated the DNA damage response (DDR) pathway in TC cell lines, with known *TP53* mutational profile and response to the TKI Lenvatinib (7).

Pharmacological approaches targeting p53 have been tested in preclinical models of TC, either by directly restoring p53 function (17, 21), or by targeting the MDM2-p53 interaction with APG115 (22). Alterations that can affect p53 function are highly variable, including loss of its expression or truncated forms, as well as genetic mutations in upstream regulators such as the histone acetyltransferase CREBBP/EP300, which promotes p53 stabilization, or in its negative regulator MDM2 (23). Therefore, we chose an indirect approach by addressing other molecules involved in the p53 activation pathway, such as the DDR. Interestingly, some key players of the DDR pathway are targets exploited for the treatment of several tumors (11, 12) but less studied in TC (24–26).

Here, we characterized the DDR pathway in a panel of TC cell lines and found a significant variability in the expression of some key proteins (ATM, CHK1, CHK2), particularly in p53-defective TC cells, indicating that tumor cells use different mechanisms to cope with the absence of p53 function (27). Moreover, p53-defective TC cell lines displayed genomic instability, as demonstrated by higher levels of γH2AX, a marker of DNA damage, compared to p53-proficient TC cells. Consistently, genomic instability in TC is known to confer increased aggressiveness to cancer cells (9, 28–30).

In p53-proficient TC cells, double strand breaks DNA damage caused by DX primarily activates the ATM-CHK2 pathway. Activated ATM phosphorylates CHK2, which stabilizes p53, leading to cell cycle arrest, DNA repair, or apoptosis. Because p53 efficiently regulates the damage response, activation of the ATR-CHK1 pathway is usually not required in response to double strand breaks. In p53-defective cells, although ATM-CHK2 is still activated, the absence of functional p53 impairs proper cell cycle control. Consequently, cells accumulate replication stress, which triggers activation of the ATR-CHK1 pathway as a compensatory mechanism when p53 function is lost (31). DX can also induce accumulation of reactive oxygen species contributing to DNA damage formation and further activation of CHK1 (32).

Therefore, CHK1 was chosen as the molecular target for this study, given that CHK1 inhibition has been shown to induce synthetic lethality in other p53-defective cancers (12, 33–35). To the best of our knowledge, CHK1 inhibitors have not yet been evaluated in TC, either *in vitro* or *in vivo*.

Among the second generation of CHK1 inhibitors, Prexasertib (PX) proved highly selective for CHK1 kinase, competing with its ATP-binding site, and is in Phase I and II clinical trials for solid and hematological tumors (12, 19).

Due to the synthetic lethality induced by the concomitant loss of function of both CHK1 and p53, we found that PX exerts a greater antiproliferative effect in p53-defective TC cell lines by inducing DNA damage accumulation. Indeed, in the absence of functional p53, these cells cannot properly activate the canonical ATM-CHK2-p53 pathway following DNA damage induced by Doxorubicin. As a result, they become highly dependent on compensatory pathways, particularly ATR-CHK1, to manage replication stress and DNA lesions. PX-mediated CHK1 inhibition removes this critical backup mechanism, preventing efficient DNA damage detection and repair and impairing cell cycle checkpoint enforcement. This leads to accumulation of unrepaired DNA lesions, increased genomic instability, and ultimately replicative catastrophe, leading to cell death.

Indeed, in some TC cell lines the CDC25c phosphatase, a downstream effector of CHK1, was inactivated (dephosphorylated) due to CHK1 inhibition by PX. Consequently, in the presence of functional p53, as in the TPC-1 cell line, PX induced the cell cycle block in G2/M phase due to p21-mediated inhibition of the pro-mitotic complex CyclinB1/CDK1, as already reported (36). Instead, in p53-defective TC cell lines where PX effectively induced CDC25c inactivation, we found replicative failure (i.e. cell cycle block in S phase) because neither the p53/p21 axis nor the CHK1/CDC25c one could prevent CyclinB1/CDK1 complex to promote G2/M checkpoint blockade to repair DNA before mitosis. This effect may eventually induce a replication-fork collapse in the following S phase, as described in other p53-defective tumors (37, 38). Due to replicative failure, TC cells died as demonstrated by increased levels of apoptotic markers, particularly in p53-defective cells, as previously proposed by King and colleagues (19).

Notably, p53-proficient IHH-4 cell line shows comparable results with the p53-defective TC cells in response to PX treatment, likely because it harbors gene mutations in p53 regulators, namely the histone acetyltransferase EP300/CREBBP involved in p53 stabilization.

The only cell line with apparent resistance to PX, B-CPAP, showed limited antiproliferative effect and partial inhibition of pCHK1(S296). It is likely that B-CPAP cells resist CHK1 inhibition by keeping low basal CHK1 activation, despite its high protein expression, because they rely on other pathways to cope with DNA damage. For instance, a CHK1-inhibitor-resistant cell line from aggressive ovarian cancer was proven to delay G2/M phase progression, thus preventing replication failure (39).

Our study discovered that p53-defective TC cell lines were particularly affected by stress sensitization. Indeed, the combination of the lowest doses of PX and DX were able to further reduce cell proliferation compared to single treatments, due to increased cell death in all p53-defective TC cells, including the PX-resistant B-CPAP cell line. These findings are in line with the findings observed in other cancers (40, 41) and are relevant for the translational purposes of our study, aiming to propose novel treatment strategies that might reduce drug doses and related side effects in advanced TC patients. Indeed, CHK1 inhibitors combined with lower doses of Gemcitabine have already shown promising results in Phase I and II clinical trials in other tumors (42, 43).

Collectively, the aim of this study was meant to explore the DDR pathway rewiring in p53-defective TC, which emerged to take advantage of CHK1 axis as a backup strategy to cope with the most dangerous form of DNA damages, the double strand breaks, in the absence of p53 functionality. This prompted a novel translational rationale for targeting CHK1 dependence in p53-altered TC and supporting further preclinical development of combined PX and DX regimens in this challenging disease context.

In conclusion, this study demonstrates for the first time that the inhibition of the DNA damage response kinase CHK1 by Prexasertib is effective against thyroid cancer cell lines with different types of p53 alterations as well as mutations in EP300/CREBBP, encoding for p53 regulators. The anti-proliferative effect of PX is primarily due to the induction of cell replicative failure and cell death at low nanomolar concentrations, following the synthetic lethality principle. Furthermore, by combining treatments with the translational aim of reducing drug doses and side effects, we show that the effect of PX on aggressive TC cell lines is potentiated by Doxorubicin in p53-defective cell lines. Thus, the evaluation of p53 functional status could help predict which patients might benefit from PX treatment. Future studies in animal models are needed to confirm these *in vitro* findings.

## Data Availability

The datasets presented in this study can be found in online repositories. The names of the repository/repositories and accession number(s) can be found below: https://doi.org/10.5281/zenodo.15370730.
