# Supplementary material for: The CHK1 inhibitor Prexasertib is effective against in vitro models of aggressive thyroid carcinomas with defective p53 function
Source: Front Endocrinol (Lausanne). 2026 May 8;17:1743111. doi: 10.3389/fendo.2026.1743111 (PMC13193820; doi:10.3389/fendo.2026.1743111)
Supplement: Supplementary file 1 [file DataSheet1.pdf]

A.

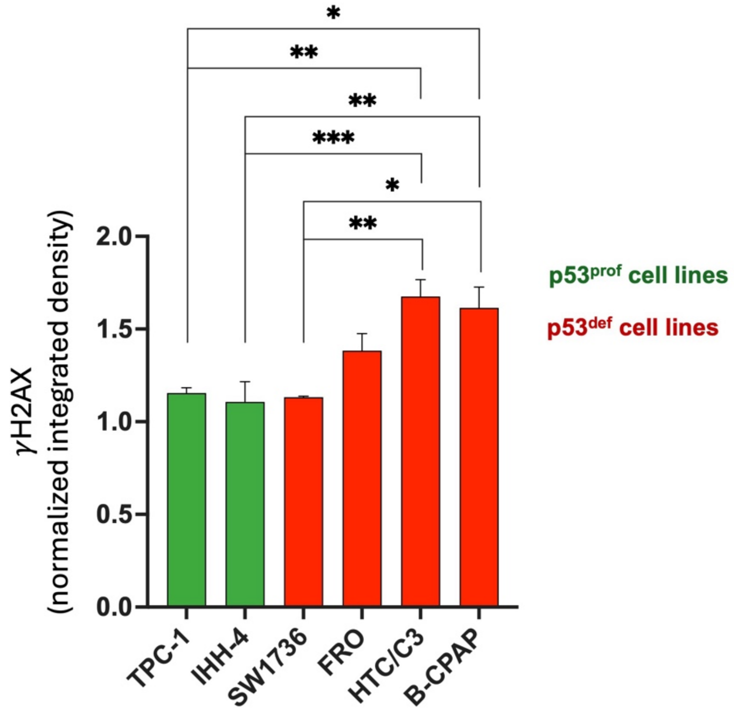

B.

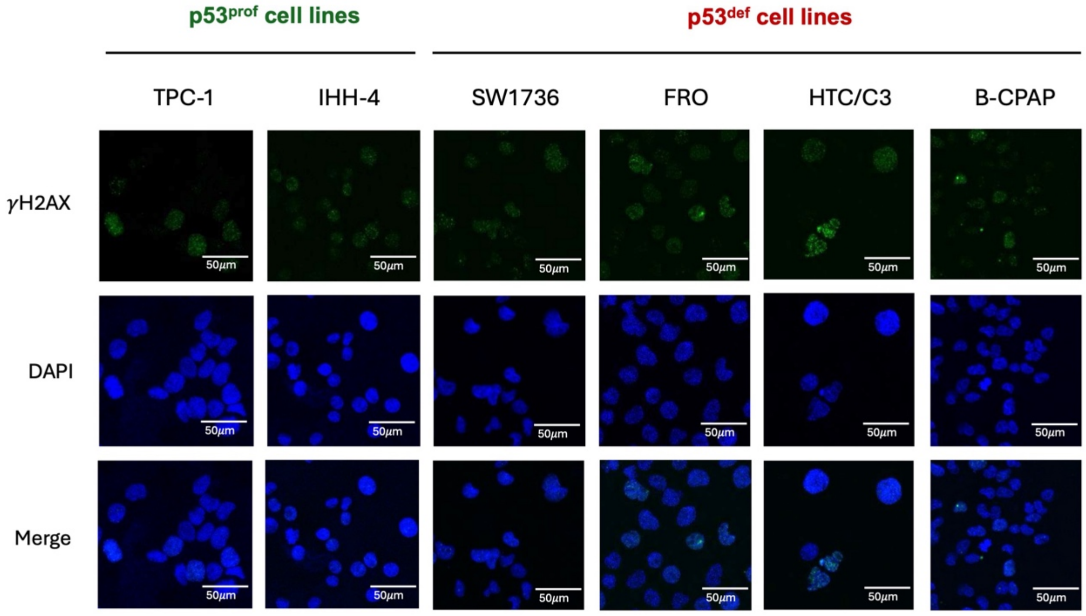

Supplementary Figure 1. TC cell lines display intrinsic DNA damage foci. A) Bar-chart showing the integrated density levels of γH2AX, marker of DNA damages, evaluated by immunofluorescence in all TC cell lines. Intrinsic DNA damage foci are more present in p53-defective TC cell lines with respect to those p53-proficient. Data are normalized on experimental endogenous control and presented as mean±SEM. The statistical significance was calculated with One-Way ANOVA test followed by Tukey's multiple comparisons test (* $p<0.05$; ** $p<0.01$; *** $p<0.001$). **B)** Representative immunofluorescence images of γH2AX (green) in TC cell lines (20X magnification). DAPI staining (blu) was used to counterstain the nucleus. Abbreviations: prof, proficient, def, defective.

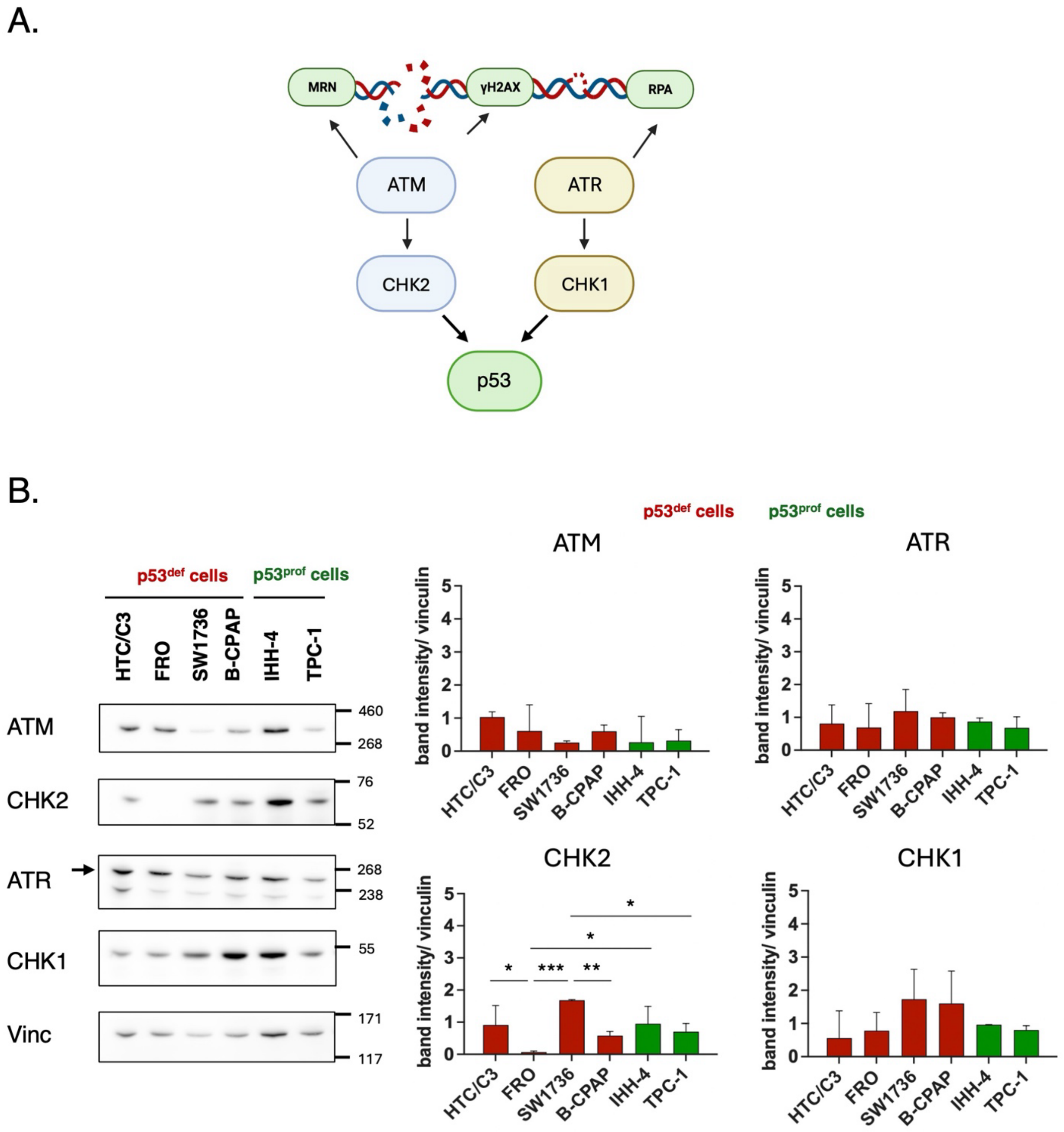

Supplementary Figure 2. DDR kinases expression is more variable in p53-defective TC cell lines. A) Scheme of DDR kinases investigated, created with https://BioRender.com (Agreement number: DE632A14-0002). B) Western blot representative images of DDR kinases ATR, CHK1, ATM, CHK2 and housekeeping Vinculin (left) and relative densitometric quantifications (right). HiMark Pre-stained Protein Standard (ThermoFisher) was used. The experiment was run in triplicate. The statistical significance was calculated with parametric One-Way ANOVA test followed by Tukey's multiple comparisons test for normally distributed data (as in the case of CHK2) expressed as mean (±SEM). The non-parametric Kruskal-Wallis test followed by Dunn's multiple comparisons test was used for skewed data (as in the case of ATM, ATR and CHK1) expressed as median ± IQR (*p <0.05; **p <0.01; ***p< 0.001). Abbreviations: Vinc, Vinculin; prof, proficient; def, defective.

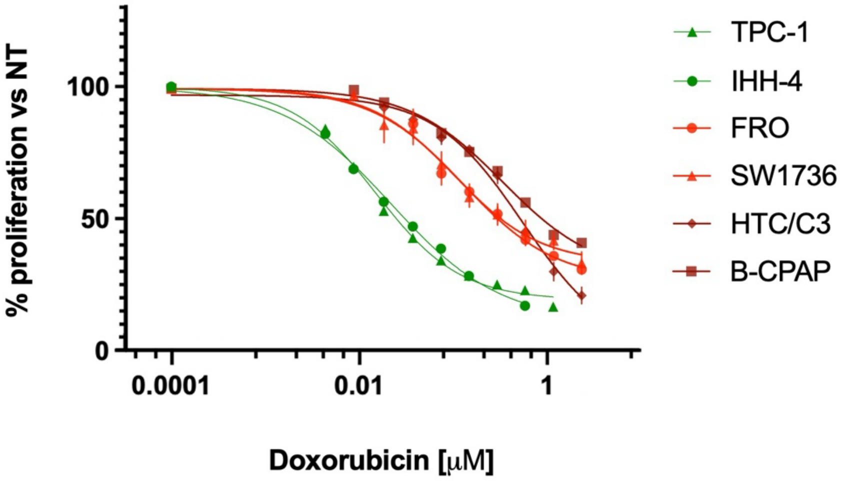

CELL LINES	IC_{25} nM	IC_{50} nM	IC_{75} nM	HISTOTYPE	p53 status
TPC-1	5.0	15.7	49.0	PTC	proficient
IHH-4	5.3	23.0	100.0	ATC	
FRO	40.0	135.0	447.0	ATC	defective
SW1736	34.0	109.0	344.0		
HTC/C3	178.0	588.0	1943.0	PDTC	
B-CPAP	99.0	362.0	1326.0		

Supplementary Figure 3. TC cell lines-specific Inhibitory Concentrations (ICs) in response to Doxorubicin. Above, dose-response curves based on the evaluation of TC cell lines proliferation treated with increasing concentrations (from 0 to 2.5μM) of Doxorubicin (DX) by MTT assay after 48 hours. The sigmoidal curves represent the proliferation trend in response to DX treatments. Below, DX Inhibitory Concentrations (ICs) values extrapolated from the dose-curves response for each cell line. The data are expressed as percentage compared to NT samples (100% of cell proliferation) and are expressed as a mean ±SEM of at least three independent experiments. Abbreviations: NT, non-treated sample; ATC, anaplastic TC; PDTC, poorly differentiated TC; PTC, papillary TC.

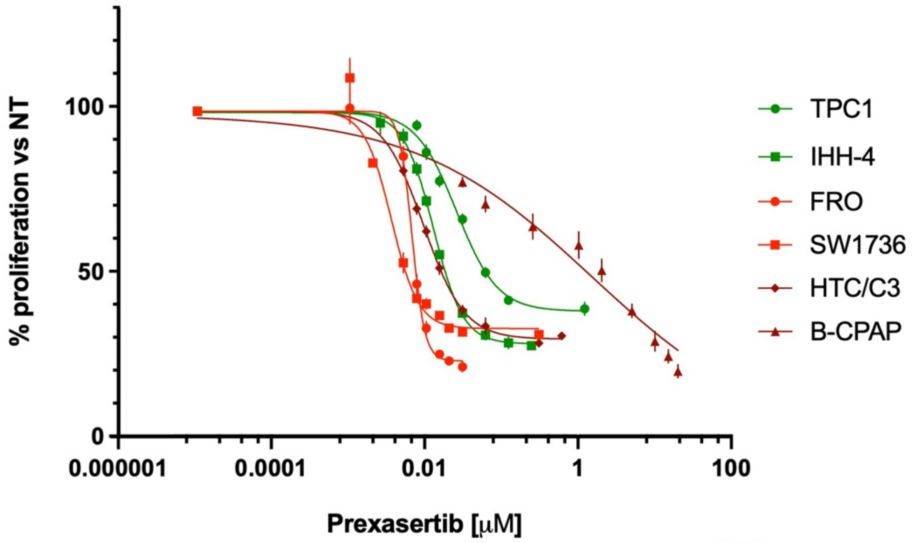

CELL LINES	IC_{10} nM	IC_{25} nM	IC_{50} nM	IC_{75} nM	IC_{100} nM	p53 status
TPC-1	4.3	7.6	13.8	25.0	40.1	proficient
IHH-4	3.9	7.6	12.4	22.0	29.1	
**MEAN**	**4.1**	**7.6**	**13.1**	**23.5**	**34.6**	
FRO	4.2	5.3	6.5	8.1	23.9	defective
SW1736	1.4	2.3	3.5	5.4	33.9	
HTC/C3	2.4	4.7	9.2	18.3	30.5	
**MEAN**	**2.7**	**4.1**	**6.4**	**10.6**	**29.4**	
B-CPAP	4.4	81.5	1490.0	2725.0	\	

Supplementary Figure 4. Dose-response curves to Prexasertib treatment. TC cells were treated with increasing concentrations of Prexasertib **(**PX) (from 0 to 20μM) and cell proliferation was tested with MTT assay after 48 hours. Above, the sigmoidal curves represent the proliferation trend in response to PX treatments. The data are expressed as percentage compared to NT samples (100% of cell proliferation) and are expressed as a mean ± SEM of at least three independent experiments. Below, Prexasertib IC values derived from dose-response curves of each cell line and mean IC values of both p53-defective (except B-CPAP) and p53-proficient cells. Abbreviations: NT, non-treated sample; IC, Inhibitory Concentrations.

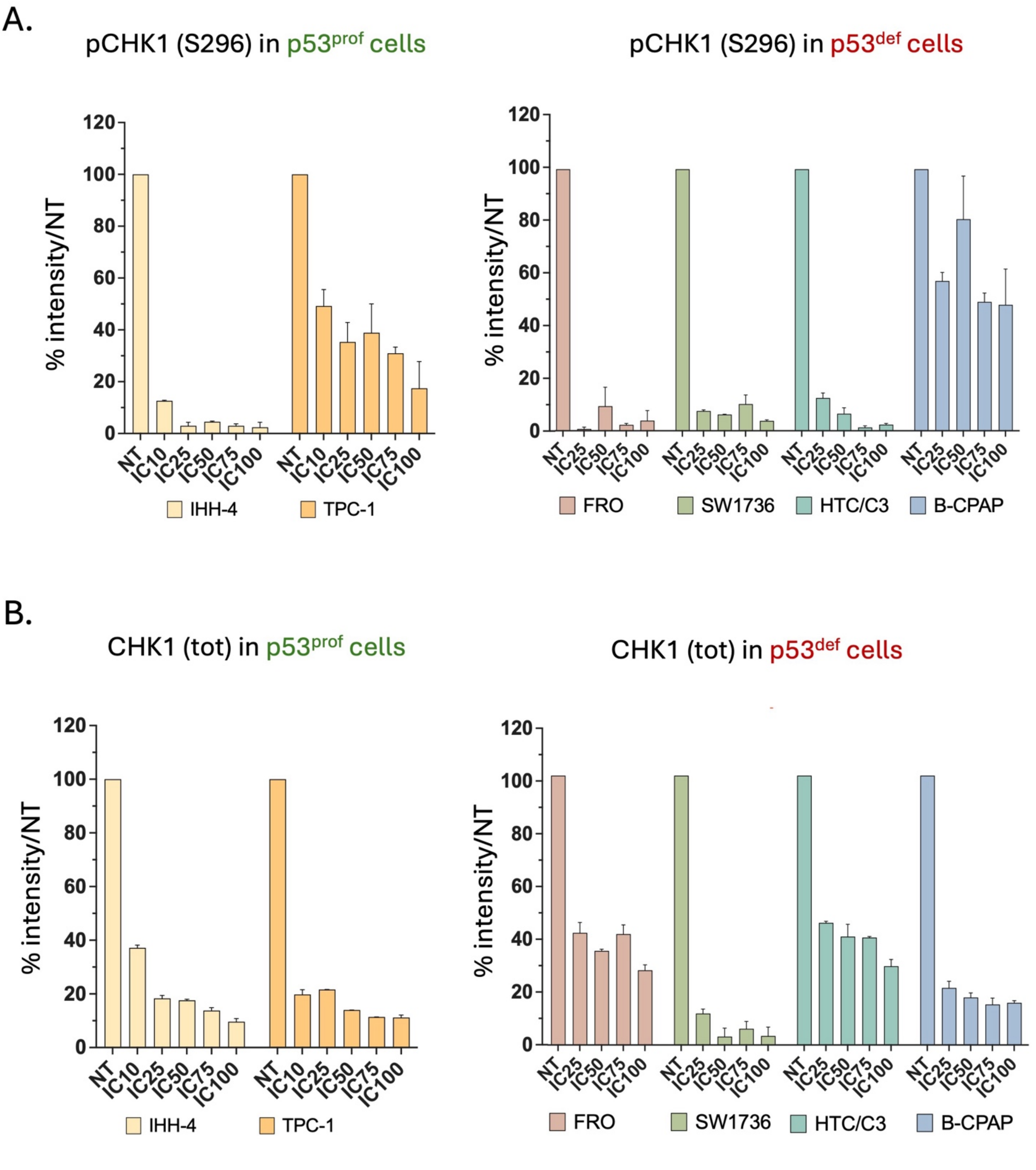

Supplementary Figure 5. PX specifically inhibits CHK1 kinase in TC cell lines. ELISA assay of phospho-CHK1 (S296) (A) and of total CHK1 protein (B) performed in TC cell lines, both p53-defective and p53-proficient, treated with mean ICs doses of PX extrapolated from dose-response curves and lysed after 48 hours. To note, p53-proficient TC cells were treated also with the IC25 value (4.1 nM) found for p53-defective TC cells, corresponding to a mean IC10. All experiments were performed once with two technical replicates. Data are reported as percentage with respect to the NT sample and expressed as median ± IQR. Abbreviations: p, phosphorylated form; PX, Prexasertib; NT: non-treated; prof, proficient; def, defective, p, phosphorylated form; tot, total-form.

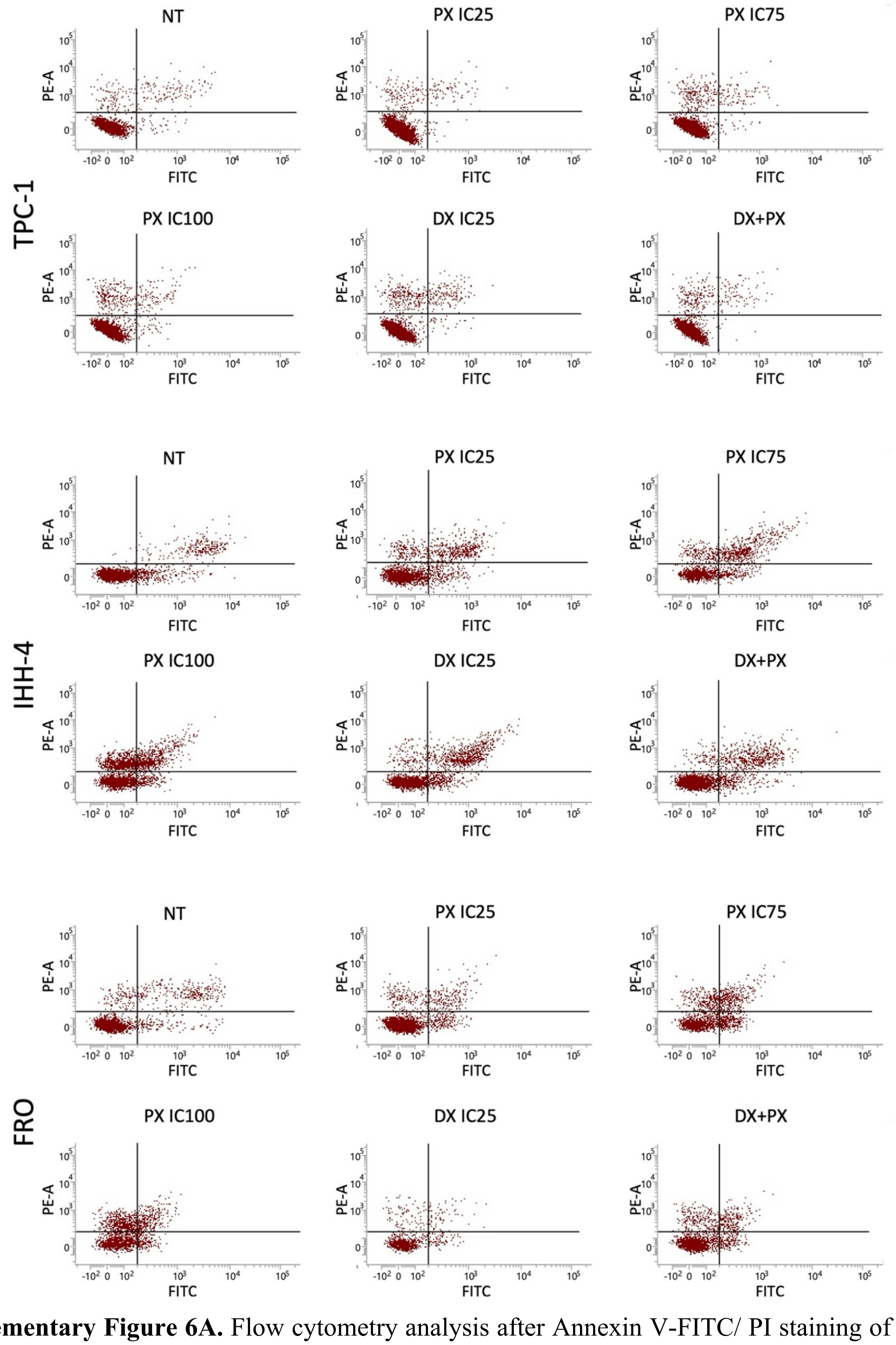

Supplementary Figure 6A. Flow cytometry analysis after Annexin V-FITC/ PI staining of TPC-1, IHH-4 and FRO cell lines. All TC cell lines were treated with mean ICs of PX, relative to each TC group of p53 functionality, DX and DX+PX. Each plot shows Annexin V-FITC fluorescence versus PI fluorescence (PE-A channel). Quadrants were set based on NT controls and applied to all samples to define viable, early apoptotic, late apoptotic cells and necrotic cells. Abbreviations: PX, Prexasertib; NT: non-treated; DX, Doxorubicin; PI: Propidium Iodide; IC: Inhibitory Concentrations.

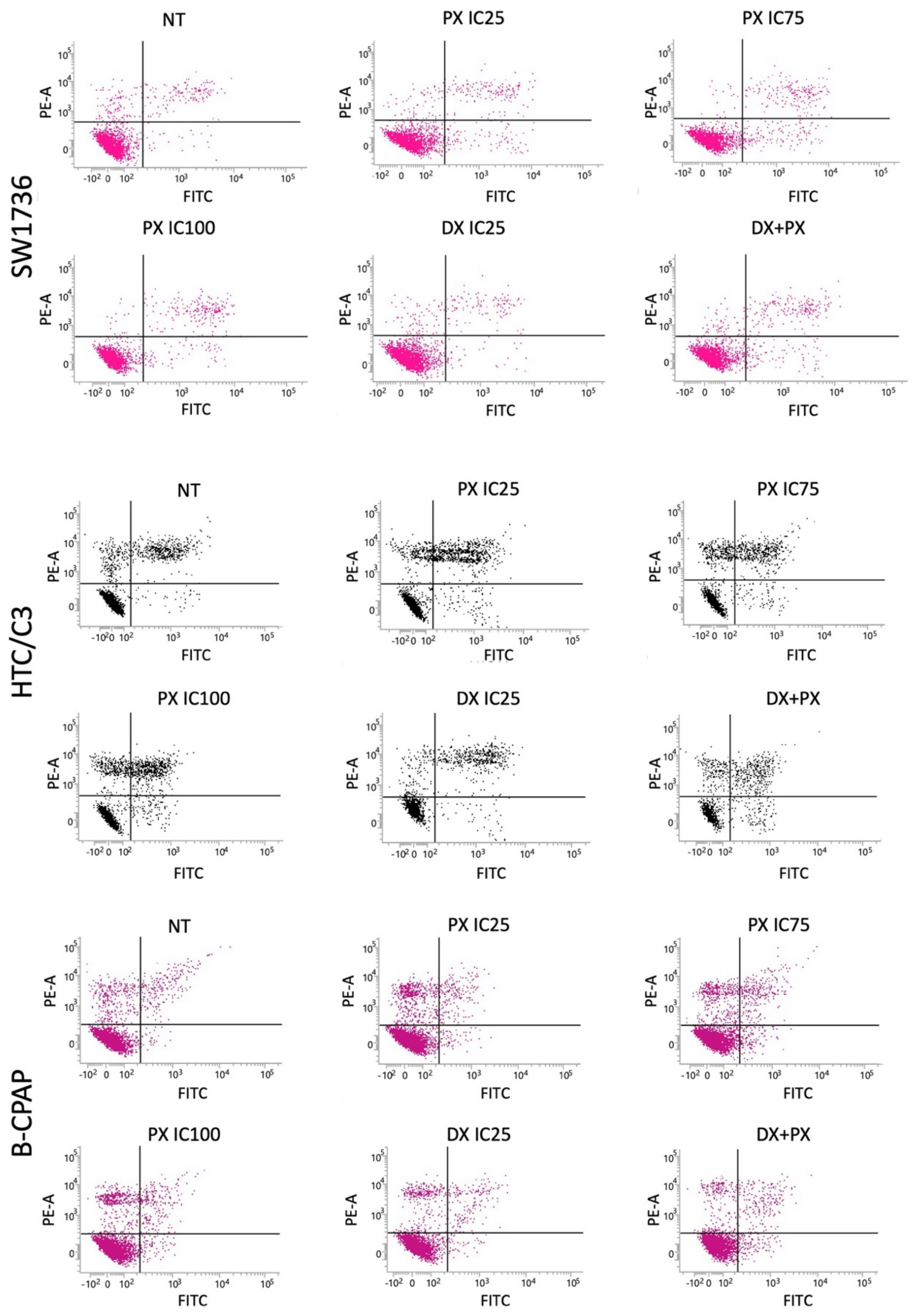

Supplementary Figure 6B. Flow cytometry analysis after Annexin V-FITC/ PI staining of SW1736, HTC/C3 and B-CPAP cell lines. All TC cell lines were treated with mean ICs of PX, relative to each TC group of p53 functionality, DX and DX+PX. Each plot shows Annexin V-FITC fluorescence versus PI fluorescence (PE-A channel). Quadrants were set based on NT controls and applied to all samples to define viable, early apoptotic, late apoptotic cells and necrotic cells. Abbreviations: PX, Prexasertib; NT: non-treated; DX, Doxorubicin; PI: Propidium Iodide; IC: Inhibitory Concentrations.
